# Localization of adenovirus morphogenesis players, together with visualization of assembly intermediates and failed products, favor a model where assembly and packaging occur concurrently at the periphery of the replication center

**DOI:** 10.1371/journal.ppat.1006320

**Published:** 2017-04-27

**Authors:** Gabriela N. Condezo, Carmen San Martín

**Affiliations:** Department of Macromolecular Structures, Centro Nacional de Biotecnología (CNB-CSIC), Madrid, Spain; University of Wisconsin-Madison, UNITED STATES

## Abstract

Adenovirus (AdV) morphogenesis is a complex process, many aspects of which remain unclear. In particular, it is not settled where in the nucleus assembly and packaging occur, and whether these processes occur in a sequential or a concerted manner. Here we use immunofluorescence and immunoelectron microscopy (immunoEM) to trace packaging factors and structural proteins at late times post infection by either wildtype virus or a delayed packaging mutant. We show that representatives of all assembly factors are present in the previously recognized peripheral replicative zone, which therefore is the AdV assembly factory. Assembly intermediates and abortive products observed in this region favor a concurrent assembly and packaging model comprising two pathways, one for capsid proteins and another one for core components. Only when both pathways are coupled by correct interaction between packaging proteins and the genome is the viral particle produced. Decoupling generates accumulation of empty capsids and unpackaged cores.

## Author summary

Viruses assemble in particular locations inside infected cells where newly replicated genomes and capsids proteins meet, called viral factories. Virus genomes are packaged inside capsids by one of two mechanisms: concerted, where a protein shell is built around the genome, or sequential, where the genome is pumped into a preformed empty shell. Although adenoviruses have been studied for more than 60 years, these two basic aspects of their biology had not been elucidated. In this work, we address these two questions by determining the location in the cell nucleus where adenovirus assembly and packaging factors meet, describing failed assembly products containing unpackaged genomes, and showing for the first time images of adenovirus capsid fragments in the process of engulfing the viral DNA. Adenoviruses can cause serious clinical problems in immunosuppressed individuals, but can also be harnessed to turn from a pathogen into a useful therapeutic tool. Understanding their biology is crucial to succeed in curing adenovirus infections, and to repurpose the virus to our advantage.

## Introduction

AdV virions consist of a 95 nm, icosahedral *pseudo*T = 25 protein shell enclosing a non-icosahedral DNA-protein core. Each capsid facet has 12 trimers of the major coat protein, hexon. A pentamer of penton base associated with a fiber trimer sits at each vertex. The most studied AdV, human AdV type 5 (Ad5), incorporates also four different minor coat proteins: IIIa, VI, VIII and IX. The AdV genome, a linear double stranded DNA molecule (~36 Kbp in Ad5), is tightly packed together with histone-like, virus-encoded proteins: core polypeptides V, VII and μ. The core also contains the terminal protein (TP) and the maturation protease (AVP) [1–3]. AdV assembly occurs in the nucleus, where hexon and penton, together with the minor coat proteins and the packaging protein L1 52/55 kDa, assemble into empty capsids. Viral genomes and core proteins are inserted into these capsids to yield noninfectious, immature particles [4]. These contain the precursor version of several capsid (pIIIa, pVI, pVIII) and core (pVII, pμ, pTP) proteins, as well as L1 52/55 kDa. Mature virions are produced upon cleavage of these precursors by AVP [5].

**Fig 1:**
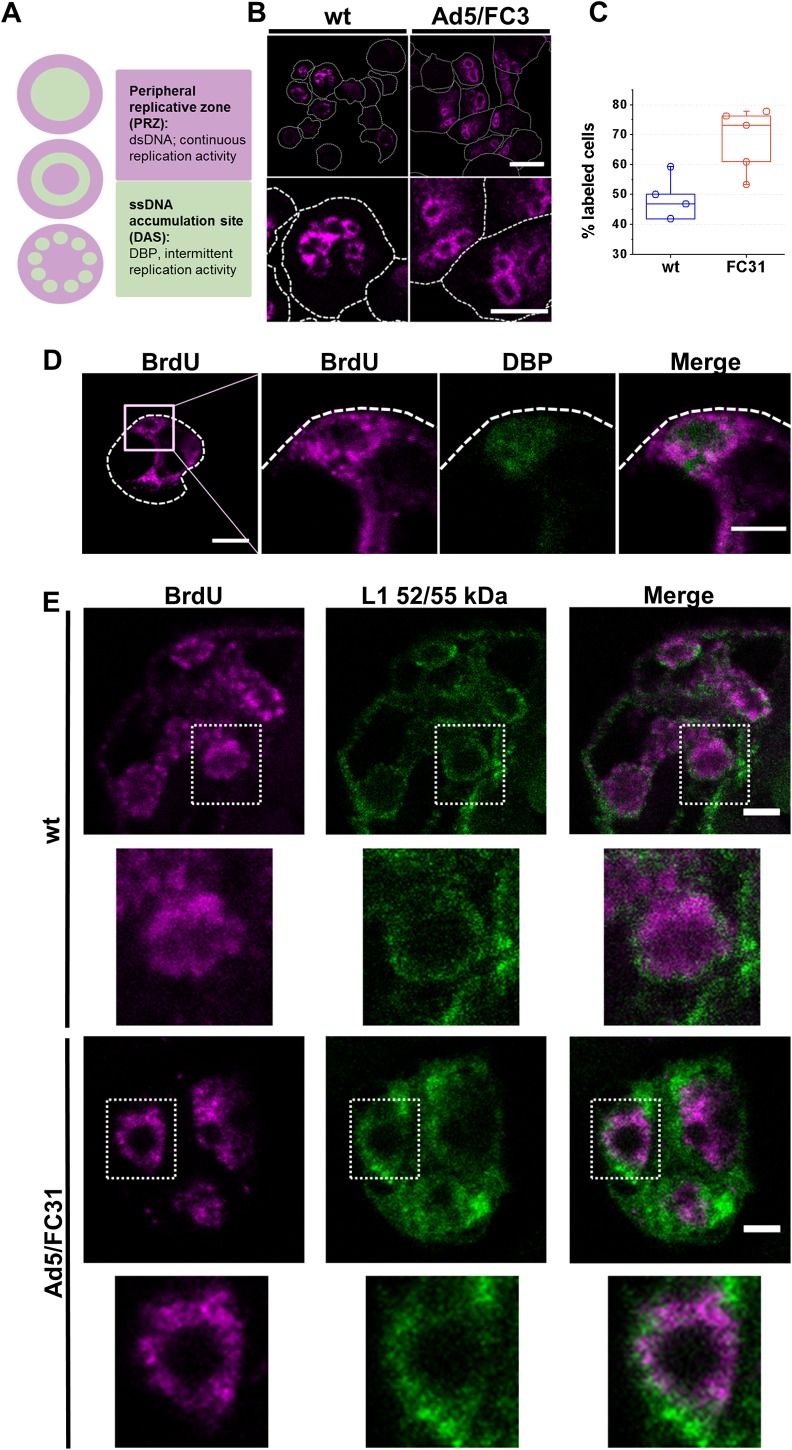
AdV genomes and packaging proteins are found together at the periphery of replication centers. **(A)** Cartoon summarizing previous observations on the distribution of AdV DNAs in the replication centers. (**B, D, E)** Confocal immunofluorescence sections (~0.3 μm thick) showing the localization of the different factors in HEK 293 cells infected with Ad5 wt or Ad5/FC31, as indicated. MOI = 50, 36 hpi. (**B)** Label for viral DNA (BrdU). The bottom row shows higher magnification views of selected areas from the top row. Scale bars: 20 μm (top row), 10 μm (bottom row). (**C**) Quantification of BrdU label. The plot depicts the percentage of infected cells (as ascertained by GFP expression) showing BrdU label. **(D)** Double labeling for BrdU (magenta) and DBP (green) in cells infected with Ad5/FC31. Bars: 10 μm (left panel), 5 μm (right panel). **(E)** Double labeling for BrdU (magenta) and L1 52/55 kDa (green). Dotted rectangles indicate the areas shown at higher magnification in the bottom row for each virus. Bars: 3 μm. In **B** and **D**, dashed white contours indicate the periphery of infected cells (assessed by GFP expression).

The Ad5 infectious cycle is completed in ~36 hours, with progeny virions appearing at 24 hours post infection (hpi). Tracing of AdV nucleic acids in infected cells has revealed where genome replication takes place [6–12]. Viral DNA is first detected (8 hpi) at the so-called early replicative sites (ERS), expanding away from nuclear domains 10 (ND10s) and containing viral ssDNA, dsDNA, and replicative activity [13]. Later on (~17 hpi), ERS evolve into two differentiated regions: the ssDNA accumulation site (DAS), harboring replicative intermediates and intermittent replicative activity, and the surrounding peripheral replicative zone (PRZ) where viral dsDNA is accumulated and there is continuous replicative activity (**Fig 1A**). Capsid proteins have been detected in a variety of AdV-induced nuclear structures, such as electron-clear inclusions (hexon, penton and polypeptide IX), electron-dense inclusions and compact rings (IVa2), or protein crystals (penton and fiber) [14–17]. However, in spite of the large amount of experimental work summarized above, it is still not clear where in the nucleus AdV assembly occurs.

The connection between capsid assembly and genome packaging is another poorly understood aspect in AdV morphogenesis. Two models have been proposed: sequential and concerted. In the sequential model, based on the dsDNA bacteriophage packaging mechanism, a motor complex would transfer the genome into a preformed capsid, using energy derived from ATP hydrolysis [18]. In the concerted model, the capsid proteins would assemble around the chromatin-like structure formed by the genome and condensing core proteins, similarly to what is thought to happen in polyomaviruses [19].

Evidence suggesting that AdV assembly is coupled to DNA synthesis would favor the concerted model. Inhibition of DNA synthesis, but not of viral proteins, results in reduced virus assembly, despite the presence of viral DNA accumulated previously to the inhibition; only DNA being synthesized is packaged into mature virions, and a thermo-conditional mutant in the AdV ssDNA binding protein (DBP) did not produce virus particles [20, 21]. DBP and ssDNA were found close to virions in cell sections, and interactions of DBP with packaging proteins have been reported [22, 23].

AdV packaging begins from the left end of the genome, where the specific packaging sequence (Ψ) is located [24]. Several virus-encoded proteins are required for genome packaging to occur: IVa2, L1 52/55 kDa, L4 22 kDa, L4 33 kDa, and IIIa. Production of empty capsids by thermo-sensitive or deletion mutants demonstrates that these proteins are required for DNA packaging but not for capsid assembly [25–30]. IVa2 and L1 52/55kDa interact during the course of AdV infection, and bind to Ψ *in vivo* independently of each other [31, 32]. Both L4 22 kDa and IVa2 bind to Ψ *in vitro*, and are required to recruit L1 52/55 kDa *in vivo* [27, 33, 34].

In genome-less AdV particles, L1 52/55 kDa forms a disordered shell beneath the icosahedral capsid, with preferential location under the vertex [35]. Interaction of L1 52/55 kDa with polypeptide IIIa, a component of the icosahedral shell located beneath the pentons, determines packaging specificity [3, 36]. Polypeptide IIIa interacts with L1 52/55 kDa *in vitro* and with Ψ *in vivo*, indicating how the genome may be tethered to the capsid during assembly. L1 52/55 kDa is released from the viral particle by proteolytic maturation, which leads to loss of interaction of this protein with itself, core and capsid proteins [35, 37]. The available evidence suggests that L1 52/55 kDa mediates the stable association between the viral DNA and the empty capsid to produce a full particle. Immature particles contain full length L1 52/55 kDa and are unable to release their genome, which stays attached to capsid fragments even under harsh *in vitro* disruption treatments [38].

Polypeptide IVa2 is thought to be present at a single vertex in the virion [39], and has Walker A and B motifs associated with ATP hydrolysis [40]. IVa2 binds ATP, and an intact Walker box is required for virion production [41], but only weak ATPase activity has been reported [42]. All these findings support the idea of IVa2 acting as the packaging motor, or a part of it, favoring the sequential assembly and packaging model. A difficulty that this model has to circumvent arises from the fact that AdV genomes are bound to cellular and/or viral proteins throughout the infectious cycle [43–47]. It is not clear how dsDNA bound to proteins would be translocated by a motor, or how the proteins would first be removed, then penetrate the capsid to reassociate with the packaged DNA. Packaging of the protein-bound AdV genome *via* an ATP driven portal would require a portal structure or mechanism different from those currently known in other viruses, or a nucleoprotein remodeling activity. Further complication is posed by the observation that the genome bound to immature core proteins is a highly compact, ~70 nm sphere [38, 48–50]. Proteins IVa2 and L1 52/55K are found both in empty capsids and bound to Ψ, suggesting that they may be present in two separate pools: a capsid-associated pool poised to receive viral DNA for encapsidation, and a second pool bound to Ψ to promote interaction between viral DNA and capsid components [36].

Another pillar supporting the sequential model is the routine appearance in AdV purifications of low density particles. These particles are considered procapsids (precursors to mature virions), because in pulse-chase experiments they appear earlier, contain protein precursors and no genome, or only fragments [4, 51–55]. The presence of different lengths of packaged DNA has also been taken as evidence for the sequential packaging model. However, the actual origin of these DNA fragments is not well understood, and an alternative origin as replication artifacts has been proposed [55, 56]. Additionally, light particles do not progress into mature virions, suggesting that they are not assembly intermediates but defective assembly products [57, 58]. This possibility is supported by the recent molecular characterization of incomplete particles produced by an Ad5 variant with delayed packaging, Ad5/FC31, showing that low density particles had started, but failed to complete packaging [35].

Ad5/FC31 was generated by insertion of the Φ31 recombinase target sequences, *attB/attP*, flanking Ψ [59]. In cells not expressing the recombinase, Ad5/FC31 viral protein and DNA production levels are similar to those of control virus, but the mutant produces negligible amounts of mature virions at 36 hpi, reaching virus yields similar (10-fold lower) than the control at only 56 hpi [35, 59]. Electrophoretic mobility shift assays suggested that nuclear proteins bound to *attB* interfered with correct interaction between packaging proteins and Ψ. As a result, packaging would be hindered until the interfering proteins are depleted [60].

Here we use immunofluorescence and immunoelectron microscopy to investigate the location in the cell of AdV packaging factors at late times post infection, and determine the location where genome encapsidation occurs. The labeling patterns of these factors, and the nuclear modifications induced by Ad5 and the delayed packaging mutant Ad5/FC31, are compared to obtain new information on the connection between assembly and packaging.

## Results

### Immunofluorescence localization of adenovirus genome packaging factors

To start defining the nuclear region where AdV packaging happens, viral genomes and packaging factors IVa2 and L1 52/55 kDa were localized by immunofluorescence microscopy in Ad5 wt or Ad5/FC31 infected cells at late times post infection. Previous work had shown a large divergence in mature virus production between control virus and Ad5/FC31 at 36 hpi [59]. Additionally, an electron microscopy (EM) survey of Epon-embedded infected cells from 24 to 56 hpi indicated that the nuclear modifications induced by both viruses during the first 24 hours were highly similar [61], while some noticeable differences (discussed later on) were found at 36 hpi and peaked at 48 hpi. Therefore, we carried out the experiments described here at 36 or 48 hpi.

Taking into account that at 18 hpi cellular DNA replication no longer occurs **(S1 Fig)** [62], two doses of BrdU were supplied to infected cells at 18 and 25 hpi, to ensure that all viral DNA synthesized at late times post-infection was labeled. BrdU presented a diffuse ring pattern, and more DNA (more labeled cells) was detected in Ad5/FC31 than in wt infections, consistent with the mutant normal replication but deficient packaging phenotype [60] (**Fig 1B and 1C**). Measurements in orthogonal views indicated that the labeled rings were in fact ellipsoids with maximum and minimum axes 6.8 ± 1.8 μm and 5.0 ± 1.2 μm (n = 40). Since this label pattern is similar to that previously reported for earlier (20–24 hpi) replication foci [9, 12], we reasoned that the diffuse ring BrdU label corresponded to the PRZ, and the unlabeled area inside the ring could correspond to the DAS (**Fig 1A**). In double labeling experiments, DBP was found adjacent to or surrounded by BrdU label (**Fig 1D**). Regions labeled for DBP but not for BrdU would correspond to ssDNA synthesized at early times post-infection, before addition of the first BrdU dose. These results indicate that BrdU labeling is revealing AdV replication centers at late times post-infection.

To investigate where packaging proteins and viral genomes meet, double labeling assays for BrdU and L1 52/55kDa, or BrdU and IVa2 (**S1 Text**), were carried out. Label for IVa2 was weak, perhaps correlating with the low copy number of the protein in the virions, or with limited antibody reactivity. L1 52/55kDa was detected at the periphery of replication centers labeled with BrdU (**Fig 1E**), where both signals intermingled. There was no label for L1 52/55 kDa in the BrdU-unlabeled areas corresponding to the DAS. The presence of both L1 52/55 kDa and viral genomes in the PRZ suggests that DNA packaging occurs in this area.

### Immunoelectron microscopy localization of adenovirus genome packaging factors

To obtain more detail on the possible packaging site of AdV, EM was used. First, sections from Epon-embedded cells were analyzed for regions in the nucleus that could correspond to PRZs. Previous studies [10, 11, 23] indicated that PRZs are ring-shaped, moderately electron-dense regions, surrounding electron-clear areas corresponding to the DAS, and containing electron-opaque granules (EOGs) and viral particles. Regions corresponding to these characteristics, and in a size range compatible with the immunofluorescence observations described above, were identified in both Ad5 wt and Ad5/FC31 infected cells (**S2 Fig)**. EOGs were often found in contact with loose electron-dense material, which was tentatively called DNA bundle because of its texture (**S2 Fig**).

**Fig 2:**
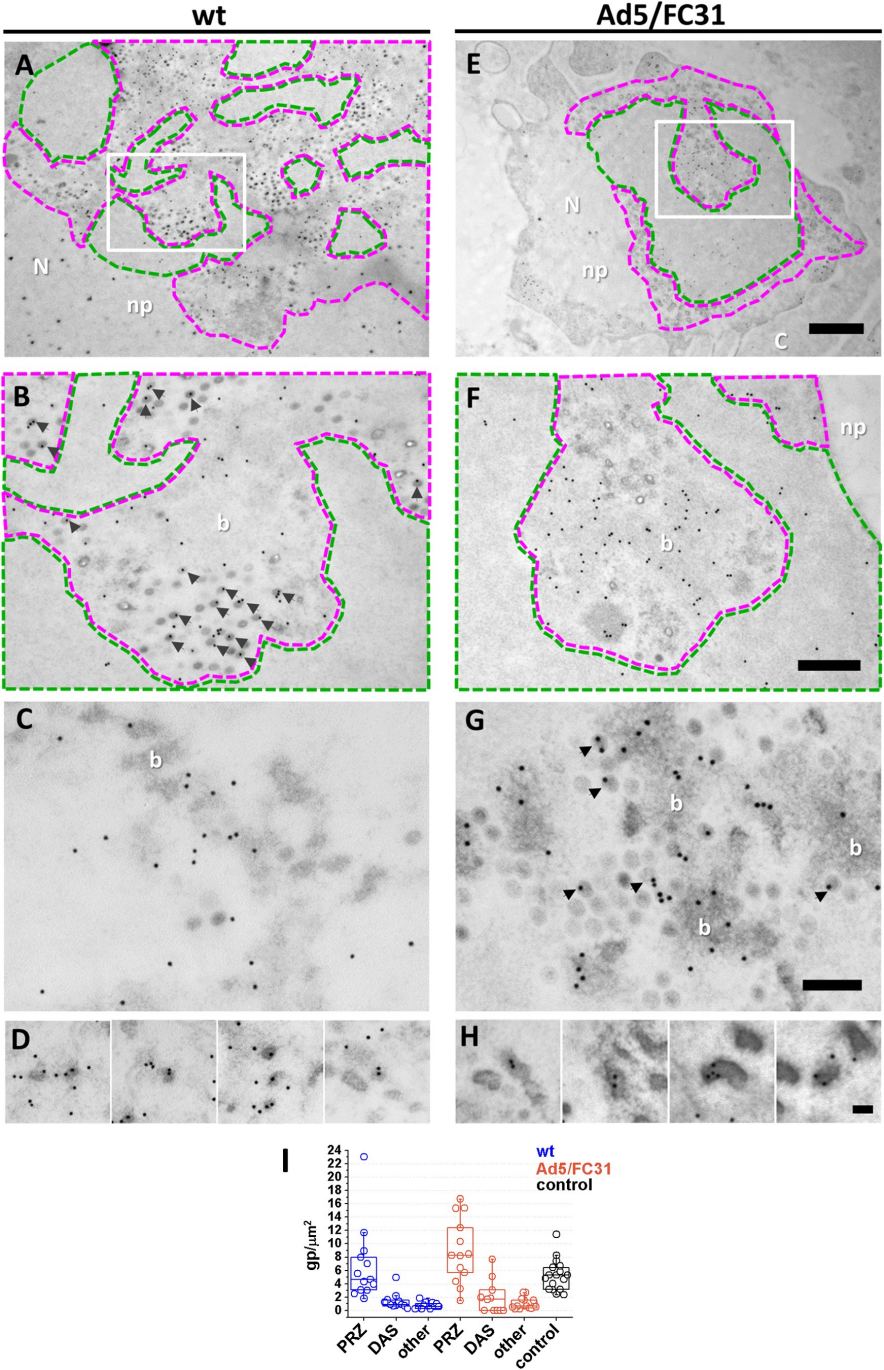
Localization of newly synthesized viral DNA in infected cells. Viral replication centers labeled with anti-BrdU in HEK293 cells infected with Ad5 wt (**A-D**) or Ad5/FC31 (**E-H**). **(A, E)** Low magnification views of replication centers with the DAS (electron-clear) highlighted in green and the PRZ in magenta. (**B, F)** Zoom of areas highlighted by white rectangles in **A** and **E**. (**C, G)** Details of other PRZs showing BrdU signal on bundles, indicating that they contain viral DNA. (**D, H)** EOGs produced by Ad5 wt and Ad5/FC31 respectively. Scale bars: 1 μm **(A and E);** 0.4 μm **(B and F);** 200 nm **(C and G)** and 100 nm **(D and H).** Nucleus (N); cytoplasm (C); DNA bundles (b); nucleoplasm (np). Arrows indicate viral particles with BrdU signal. Unless stated otherwise, all EM images correspond to 48 hpi at MOI = 50. **(I)** Quantification of BrdU label in FS samples. The number of gold particles (**gp**) per unit area (**μm**^**2**^) is shown for three different nuclear regions: **PRZ**, **DAS**, rest of nucleus (**other**). Label in **control**, uninfected cells is also shown.

To corroborate the identity of the possible PRZs, infected cells were treated with BrdU and processed for immunoEM by freeze substitution (FS), to preserve both structure and immunoreactivity. In FS samples, the possible PRZs and DAS regions were identified on the basis of their electron-density and the presence of EOGs and viral particles (**Fig 2A and 2E**). Label for BrdU was specifically found in the possible PRZ area, confirming its identity (**Fig 2B, 2F and 2I**), and was frequently associated to full particles (virions) (**Fig 2B and 2G**), EOGs (**Fig 2D and 2H**) and the loose electron-dense material (“bundles”, **Fig 2B, 2C, 2F and 2G**), confirming that they contain viral DNA. The different electron-density levels of these structures are suggestive of different degrees of DNA condensation, from most relaxed (bundles) to most condensed (virions and EOGs). The BrdU signal in EOGs indicates the presence of viral DNA, and not only RNA as reported by other studies [63]. EOGs had various sizes but they were generally larger than viral particles. No significant BrdU label was observed in other nuclear regions or in the cytosol. The electron-clear area proposed to be the DAS showed weak label (**Fig 2B and 2F**), in agreement with the immunofluorescence results and previous reports indicating low replicative activity in this region. Labeling with anti-DBP antibody was unsuccessful, suggesting that even in FS conditions the reactivity of the DBP epitopes was not preserved.

**Fig 3:**
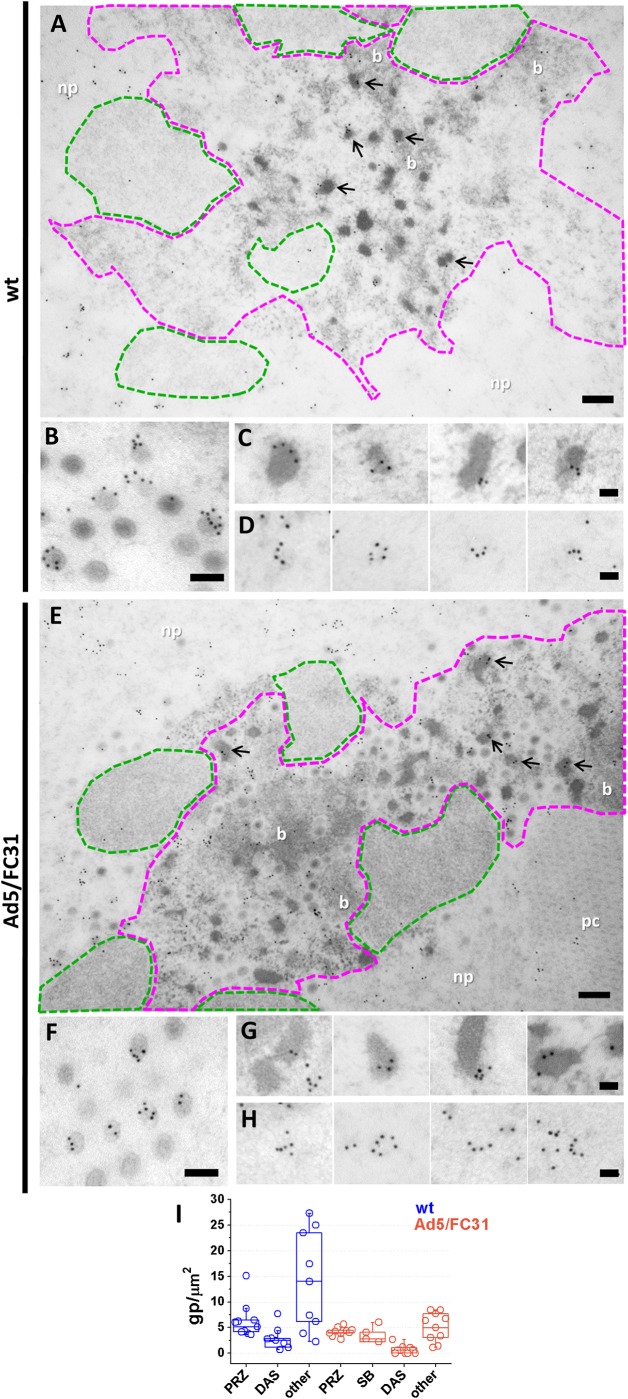
Localization of protein L1 52/55 kDa by immunoelectron microscopy. HEK 293 cells infected with Ad5 wt (**A-D**) or Ad5/FC31 (**E-H**) labeled for L1 52/55 kDa. (**A, E)** General view of replication centers. DAS area highlighted in green and PRZ in magenta. Arrows indicate the presence of L1 52/55 kDa in EOGs. (**B, F)** Viral particles. **(C, G)** Electron-opaque grains. (**D, H)** Arch-shaped labels in the nucleoplasm close to the PRZs. Nucleoplasm (np); protein crystal (pc); DNA bundle (b). Scale bars: **A and E** 200 nm; **B and F** 100 nm; **C-D and G-H** 50 nm. **(I)** Quantification of gold labels for L1 52/55k in the different nuclear regions in the infected cell. SB refers to speckled bodies, introduced later on in the text.

Next, the localization of packaging proteins was analyzed. Scattered label for L1 52/55 kDa was observed throughout the infected nuclei, including the PRZ (**Fig 3A, 3E and 3I**). However, very few gold particles were observed in the DAS, supporting the specificity of the label (**Fig 3I** and **S1 Table**. L1 52/55 kDa signal in the PRZ was usually associated to the electron-dense, BrdU-positive features present in this area (EOGs and bundles, **Fig 3**). As expected, L1 52/55 kDa was detected in viral particles, particularly in those with lower electron density indicating incomplete packaging (**Fig 3B and 3F**). Label in viral particles often presented an arch pattern consistent with a shell of this protein inside the capsid [35]. Arch patterns were also found in EOGs (**Fig 3C and 3G**), suggesting the formation of an L1 52/55 kDa shell on the electron dense material they contain. These observations are consistent with the presence of L1 52/55 kDa in two pools, one binding to the viral DNA (bundles and EOGs) and another binding to capsid proteins (electron-clear capsids) [36]. Interestingly, groups of gold particles also forming little arches were frequently found near the PRZ (**Fig 3D and 3H**), suggesting L1 52/55 kDa shell fragments on their way to assemble with viral genomes or capsid proteins. This interpretation is consistent with the previously reported homo-oligomerization capacity of L1 52/55 kDa [37, 64].

Label for packaging protein IVa2 was weak, as previously observed in immunofluorescence (**S1 Text**). Nevertheless, signal for IVa2 was present in the PRZ on electron-dense material (bundles and some EOGs) (**S4 Fig** and **S1 Table**). Summarizing, immunoEM confirmed that AdV genomes and packaging proteins are present in the PRZ. Genomes were present in the form of loose bundles and EOGs that by their electron density, texture and label for BrdU and packaging proteins are consistent with condensed viral genomes. PRZs also contained viral particles. These results suggest that the PRZ could be the location in the nucleus where AdV genome encapsidation takes place.

### Immunoelectron microscopy localization of adenovirus structural proteins

**Fig 4:**
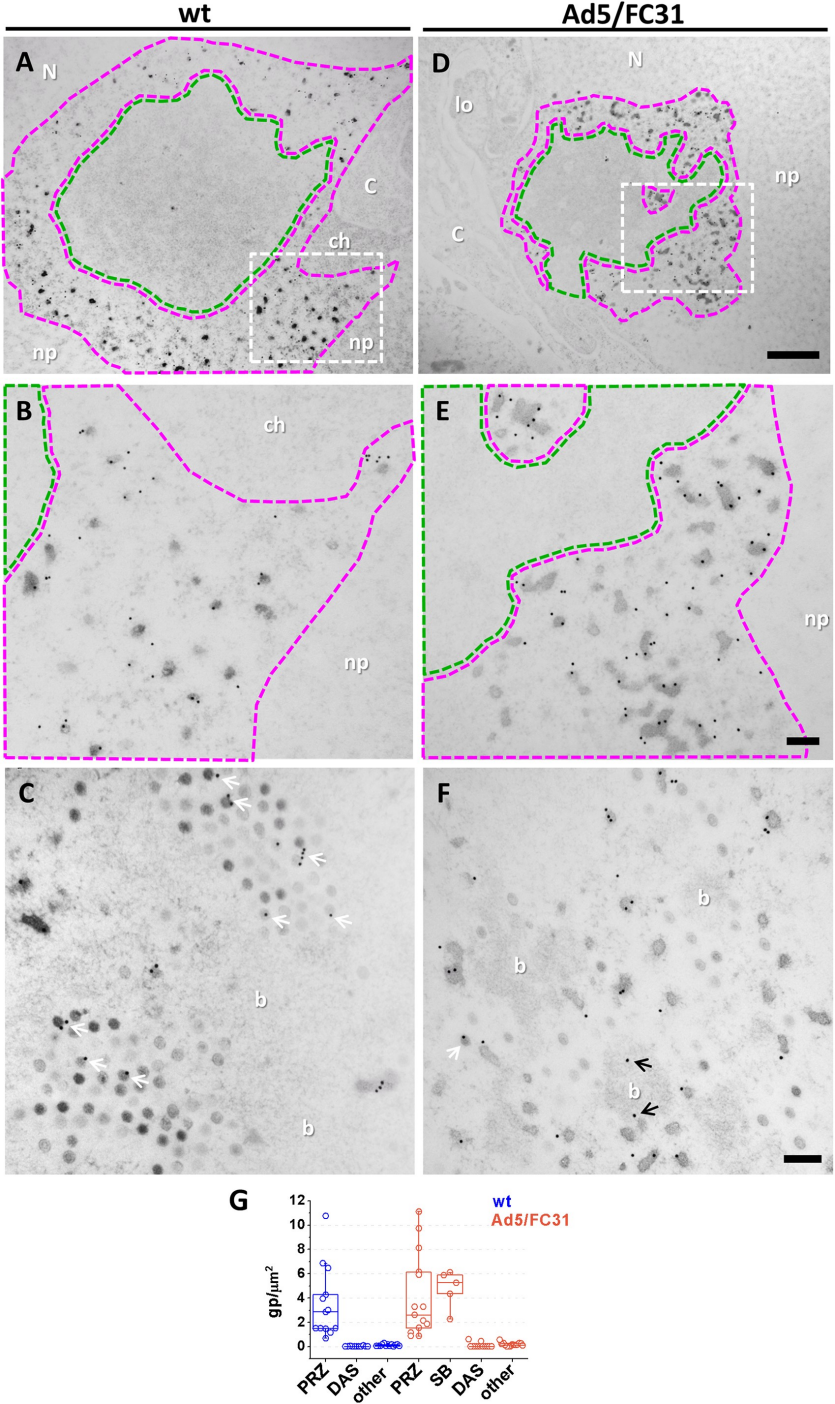
Presence of core protein VII in the putative AdV assembly zone. Replication centers labeled for protein VII in HEK293 cells infected with Ad5 wt **(A-C)** or Ad5/FC31 **(D-F)**. Sections treated with DNase before immunolabeling to unmask VII epitopes. **(B, E)** Zoom of square areas in **A, D**. Green area: DAS; magenta area: PRZ. White arrows indicate signal in viral particles; black arrows indicate signal in DNA bundles. Nucleoplasm (np); nucleus (N); cytoplasm (C); chromatin (ch); DNA bundle (b); lobes (lo). Scale bars: **A and D** 1 μm; **B, C, E and F** 0.2 μm. **(G)** Quantification of label for protein VII in FS samples.

To further assess the hypothesis that AdV assembly occurs at the PRZ, the presence of core (VII) and capsid (fiber) proteins was analyzed. Label for VII was exclusively observed in the PRZ, frequently in EOGs (**Fig 4**) and in lower amounts in DNA bundles (**Fig 4F**), corroborating the idea that they contain viral DNA condensed to different degrees by core proteins. Protein VII was also detected in viral particles (**Fig 4C**).

**Fig 5:**
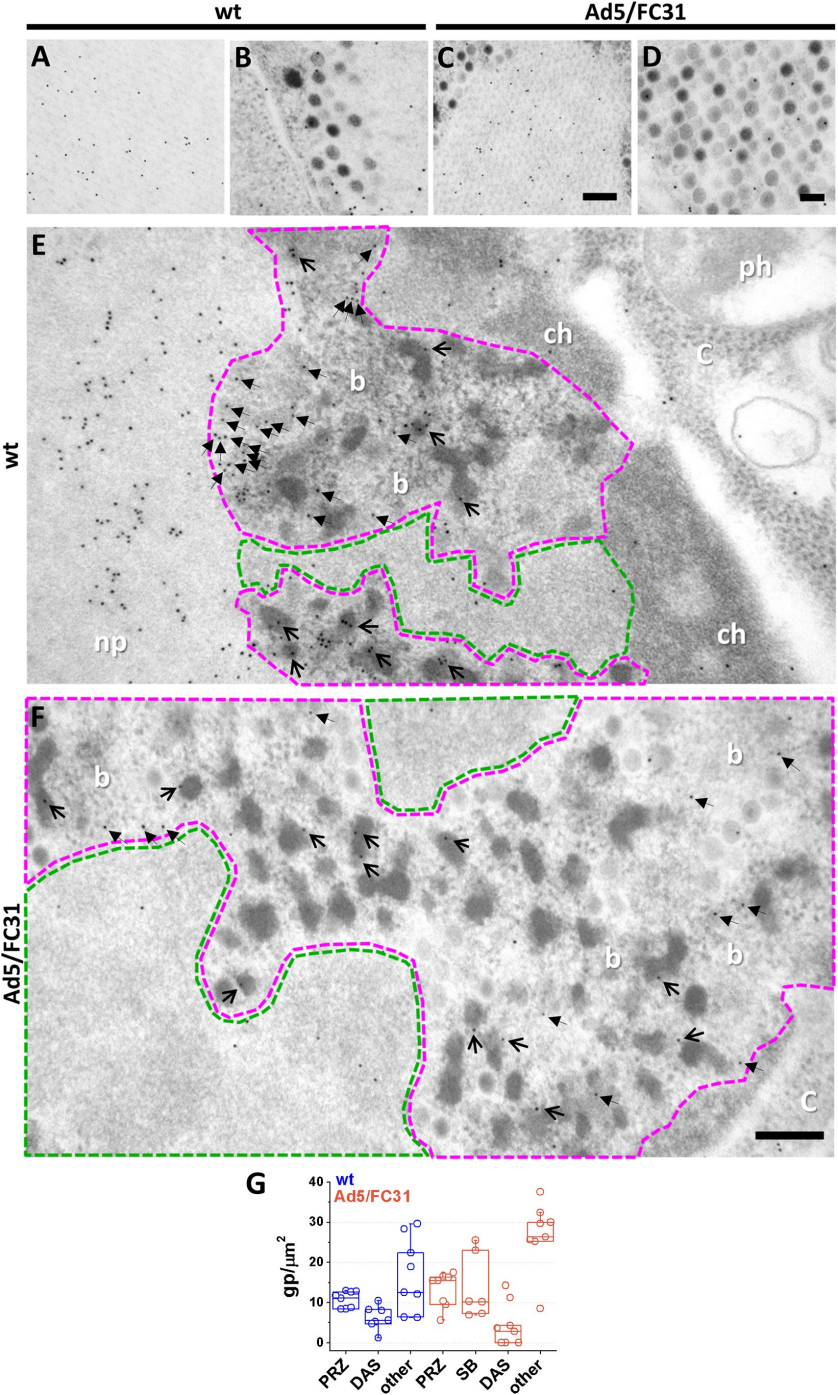
Presence of capsid protein (fiber) in the putative AdV assembly zone. HEK 293 cells infected with Ad5 wt **(A, B, E)** or Ad5/FC31 **(C, D, F)** labeled for fiber. (**A, C)** Protein crystals. (**B, D)** Viral particles. **(E, F)** Replication centers. Green area: DAS. Magenta area: PRZ. Cytoplasm (C); nucleoplasm (np); chromatin (ch); phagocytic vacuoles (ph); DNA bundles (b). Open and closed arrows indicate signal in electron-opaque grains and DNA bundles respectively. Scale bars: 200 nm. **(G)** Quantification of label for fiber in FS samples.

Analyzing capsid protein localization in AdV infected cells is not straightforward, since they are produced in large excess [65]. Antibodies against fiber labeled protein crystals (**Fig 5A and 5C**) and viral particles (**Fig 5B and 5D**), as expected. Fiber was also detected in the PRZ (**Fig 5E and 5F**), in particular in EOGs and bundles, but only a weak signal was observed in the DAS, supporting the specificity of the label (**Fig 5G** and **S1 Table**). The presence of core and capsid proteins, together with viral DNA and packaging factors, as well as viral particles, indicates that the PRZ is the AdV assembly site, *i*.*e*. the AdV assembly factory.

### Adenovirus assembly intermediates

**Fig 6:**
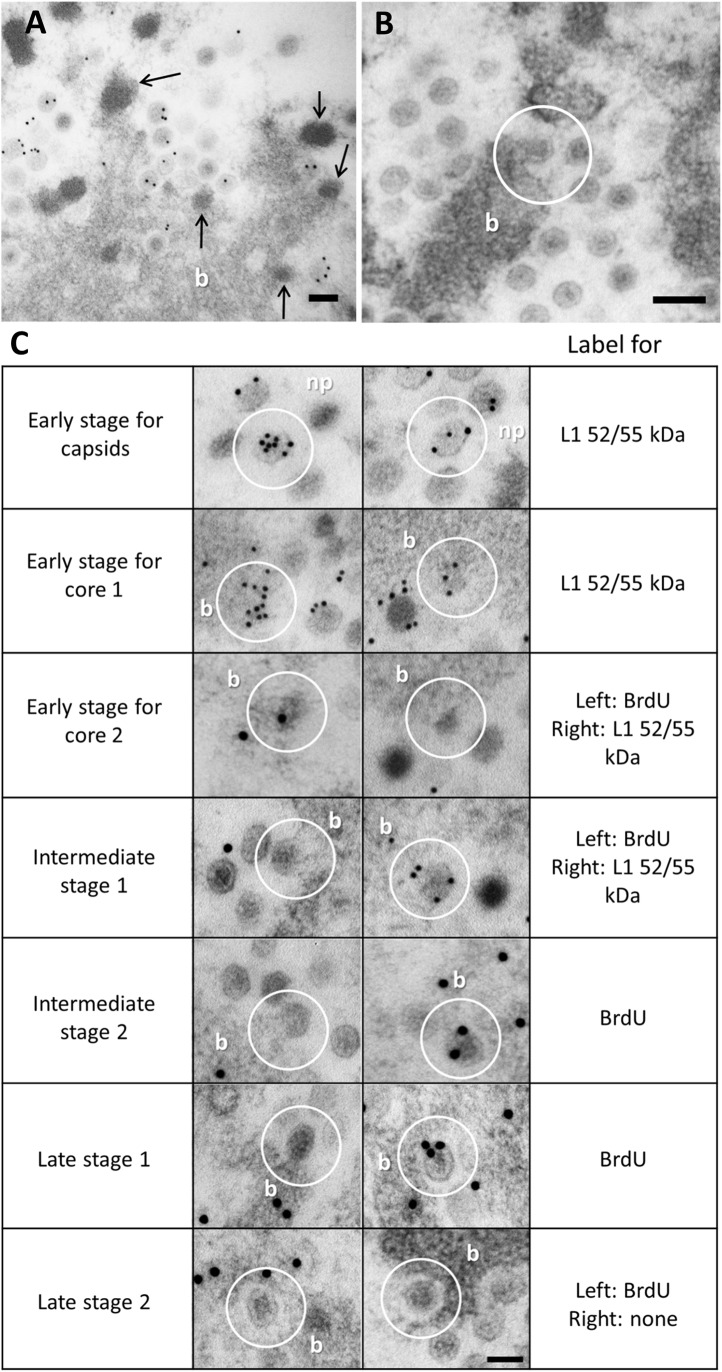
AdV assembly site and possible sequence of events. Selected details from the PRZ region in freeze-substituted HEK293 cells infected with Ad5/FC31 show where and how AdV capsid and core meet. (**A)** EOGs (arrows) arise from DNA bundles (b). Section labeled for L1 52/55 kDa. (**B)** A partially formed capsid engulfs a protrusion in a DNA bundle (white circle)**. (C)** Possible sequence of events to assemble a viral particle. White circles indicate the specific detail the text refers to. In the e*arly stage for capsids*, capsid caps are assembled in the nucleoplasm (np). These caps contain L1 52/55 kDa, which will act as a Velcro to bind to other L1 52/55 kDa molecules or other packaging elements in the core. In the *early stage for core 1 and 2*, small condensations appear at the periphery of the DNA bundle (b), also containing L1 52/55 kDa. In the *intermediate stage 1*, the capsid caps bind to these condensations through L1 52/55kDa, and capsids grow around the coalescing core (*intermediate stage 2*). In the *late stage 1*, the capsid is almost complete and the core is still connected to the DNA bundle. Finally (*late stage 2*), the viral particle is sealed and separated from the bundle. Sections labeled against L1 52/55 kDa or BrdU, as indicated. The scale bars represent 100 nm.

After determining that representatives of all AdV morphogenesis players (genome, packaging factors, core and capsid proteins) were present in the PRZ, we addressed the question of where exactly in this region was assembly taking place. Both EOGs and bundles were positive for all tested assembly factors, and both viral particles and EOGs were often found at the DNA bundle periphery, suggesting a topological relation (**Fig 6A**). Detailed observation of FS samples revealed half capsids engulfing DNA condensations protruding from the bundles (**Fig 6B**), indicating that these are most likely the assembly sites. These assembly intermediates were extremely infrequent in Ad5 wt infections, and very hard to find in Ad5/FC31: only 8 (Ad5 wt) and 25 (Ad5/FC31) such intermediates were found in a dataset consisting of 19 cells and a total scanned area of over 1600 μm2 for each virus. This observation indicates that AdV assembly is a highly cooperative process.

Exhaustive examination of all FS samples yielded a possible sequence of events in AdV assembly. On the one hand, capsid fragments are assembled containing L1 52/55 kDa (**Fig 6C, early stage for capsids**). On the other, L1 52/55 kDa also appears at the periphery of DNA bundles, often in small electron-dense protrusions suggesting the condensing action of core proteins (**Fig 6C, early stage for core**). We propose that these protrusions are nascent viral cores, containing one of the two L1 52/55 kDa pools (the one bound to the packaging sequence), and serving as the recruitment spot for the other L1 52/55 kDa pool (the one bound to capsid fragments). Incoming capsid fragments assemble around the coalescing core (**Fig 6C, intermediate stage 1**), and gradually grow (**Fig 6C, intermediate stage 2**) until the complete particle is formed (**Fig 6C, late stage 1**) and finally detaches from the DNA bundle (**Fig 6C, late stage 2**). Although EM images from cell sections are static snapshots, the observation for the first time of AdV capsids assembling around nascent cores is strongly suggestive of a concerted rather than sequential assembly and packaging mechanism.

### Adenovirus failed assembly products

Empty capsids generated by AdV mutants with packaging defects are failed assembly products made by capsid components [25–29, 35]. However, the fate of unused core components in packaging defective mutants is not known. We show here that EOGs arise from viral DNA bundles, and are labeled for structural and packaging factors, but adopt variable shapes and sizes different from those expected for a viral particle (**Fig 6A**). EOGs can therefore be interpreted as a different class of failed assembly events: cores whose association with capsid fragments was unsuccessful. In agreement with this hypothesis, we observed that EOGs were more abundant in Ad5/FC31 than in wt infections (**Figs 3A and 3E; 4B and 4E; S5 Fig** and **S1 Table**).

**Fig 7:**
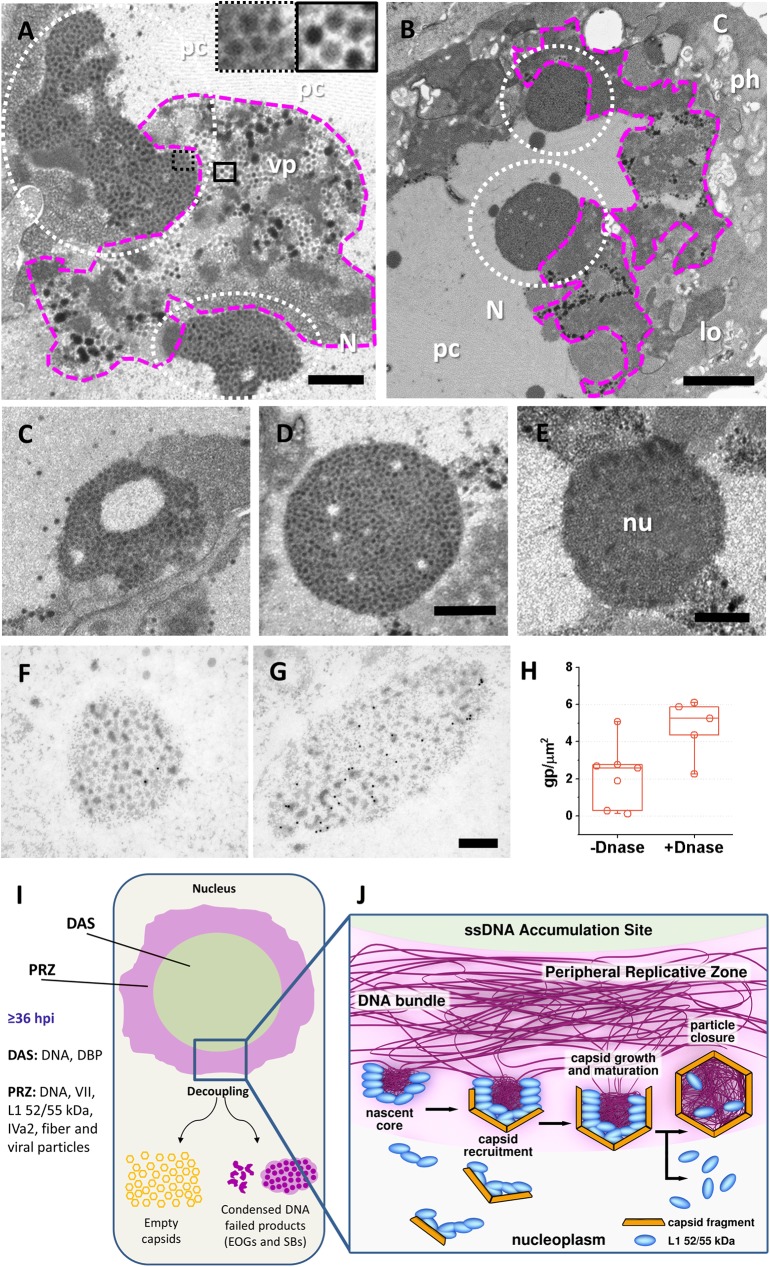
Failed core assembly products and schematics for the proposed AdV assembly pathway. **(A–D)** Cells infected with Ad5/FC31 (MOI = 5) and embedded in Epon. (**A)** Loose speckled bodies (white dotted circles) adjacent to the PRZ (magenta contour) at 48 hpi. The insets show zooms into a speckled body (dotted black square) and viral particles (black square). (**B)** Compact speckled bodies adjacent to the PRZ at 56 hpi. (**C-D)** Speckled bodies found at 36 and 56 hpi respectively. (**E)** A nucleolus in a cell infected with Ad5 wt at 48 hpi is shown for comparison. **(F and G)** Speckled bodies in freeze-substituted cells labeled for core protein VII, without (**F)** or with **(G)** DNase treatment. Nucleus (N); cytoplasm (C); virus particles (vp); lobe (lo); protein crystal (pc); phagocytic vacuoles (ph). Scale bars: **A** 700 nm; **B** 1.5 μm; **C-E** 500 nm; **F, G** 300 nm. **(H)** Quantitative comparison of polypeptide VII label in SBs with or without DNase treatment of the sections. (**I)** Schematics of an infected nucleus summarizing the localization of assembly factors in the AdV-induced structures discussed in this work. Only when interactions between packaging proteins and the viral genome are correctly coordinated, virions are assembled in the PRZ (**J**). Defective interactions give rise to dead end products: empty capsids and EOGs/SBs.

Apart from the amount of EOGs and light particles, the most remarkable difference found between Ad5/FC31 and wt infected cells was a new structure consisting of dots with very high electron density embedded in a high electron-dense background, that we named “speckled body” (SB) due to its appearance (**Fig 7**). The possibility that SBs were compact nucleoli was ruled out, because the speckles (70 ± 11 nm, n = 50) are larger than nucleoli dots (ribosomes, 25–30 nm) (**Fig 7E**), and similar in size to viral particles (**Fig 7A**, insets).

SBs were observed in Ad5/FC31 infected cells from 36 hpi, and their presence was most noticeable at 48 hpi, when they had a lobular, loose organization (**Fig 7A**), while at later times they appeared more compact and circular (**Fig 7B and 7D**). Extensive search revealed that SBs were also present in Ad5 wt infected cells, but their occurrence was extremely rare: only 2 SBs were found in a sample of 36 Ad5 wt infected cells, while 14 SBs were found in 45 Ad5/FC31 infected cells, giving a 5.5% *vs* 31.1% probability of finding a SB in an infected cell. Because of their size and texture, the speckles in the SBs are reminiscent of viral cores **(Fig 7A, insets)** [48]. SBs were often located adjacent to the PRZ (**Fig 7A and 7B**). We therefore hypothesized that SBs could be PRZ regions containing viral condensed genomes that had not been packaged due to the Ad5/FC31 mutation.

To assess the hypothesis that SBs contain condensed AdV genomes, the presence of two core components (DNA and protein VII) was tested. In initial immunolabeling experiments, no signal was observed for BrdU (viral DNA), and the signal for VII was low (**Fig 7F**). Because there was a possibility that the VII epitopes were masked by the tight complex between the condensing protein and DNA, sections were treated with DNase before incubation with the anti-VII antibody. This treatment increased the signal for VII (**Fig 7G and 7H**), indicating not only that SBs contain VII, but also that they contain DNA, and therefore viral cores. The lack of BrdU signal would indicate that the DNA in SBs was produced in the first 18 hpi, prior to incorporation of BrdU. Label for L1 52/55 kDa and fiber in SBs was comparable to that in PRZs (**Figs 3I and 5G**, and **S1 Table**). Some SBs had a ring shape with an electron-clear center reminiscent of the DAS (**Fig 7C**), suggesting that they could be early collapsed PRZs. However, no viral particles (neither empty nor full) were found in SBs. We conclude therefore that SBs are early PRZs, where capsid to core recruitment started, but failed due to the Ad5/FC31 mutation that interferes with the interaction between packaging proteins and Ψ. As a result, assembly failed to proceed.

## Discussion

AdV packaging (genome, IVa2, L1 52/55k) and assembly (core and capsid proteins) factors meet at the PRZ, where virus particles are also found (**Fig 7I**). Importantly, viral genomes and core protein VII were *exclusively* localized at the PRZ. These observations indicate that the PRZ is the AdV assembly factory, and not only the DNA replication zone as previously described [10, 11]. This localization of the AdV assembly site is consistent with previous evidence indicating that replication and assembly are coupled, and therefore should happen in the same place [20]. Our results may seem contradictory with previous studies concluding that packaging protein L1 52/55 kDa is not present at replication centers [12, 64]. The use of different labeling probes (for DBP or nucleolar proteins *vs* viral genomes) or strategies (pulse *vs* prolonged deoxyuridine incubation, cell extraction *vs* FS) may be responsible for this discrepancy.

EM images of assembly intermediates in infected cells that show capsids growing around cores provide new evidence to support the concerted assembly and packaging model. Putting together previous evidence [35–37] and the results presented here, we propose the following model for AdV morphogenesis (**Fig 7J**). On the one hand, packaging proteins bind to Ψ in the DNA bundles produced during genome replication. At the periphery of the bundles, viral genomes start to condense by the action of core proteins. On the other hand, in areas close to the PRZ, full length L1 52/55k would bind to IIIa in icosahedral shell fragments. The two pools of L1 52/55 kDa (in capsid fragments and nascent cores) interact and act as a Velcro to recruit and tether the condensing core to the nascent capsid. Capsid growth proceeds by addition of capsomers or other fragments around the core, while simultaneously L1 52/55k is cleaved by AVP and removed from the particle before capsid closure and disengagement from the DNA bundle. Changes in Ψ or the packaging proteins that impair their interaction with each other would hinder these processes at different points, resulting in genome-less particles with different degrees of maturation cleavages and abandoned cores that would eventually coalesce into EOGs and SBs. Like empty particles, EOGs and SBs are more abundant in Ad5/FC31 than in Ad5 wt infections.

Our observations and proposed assembly model are in remarkable agreement with recent computational predictions on the assembly of protein shells around a simultaneously coalescing multiparticulate cargo, which in this case would be the AdV core organized in nucleosome-like DNA-protein units [1, 66]. A delicate balance between shell-shell, cargo-cargo, and shell-cargo interactions is required for correct assembly of full particles **(Fig 7I)**. Mutations that impair the network of interactions established by packaging proteins with both capsid and genome upset this balance and lead to abortive assembly products. In particular, the simulations in [66] predict that when shell-core interactions are diminished, only a few cargo molecules will be captured, leading to nearly empty shells. Notably, these empty shells can be completely closed, explaining a puzzling occurrence previously observed for the genome-less particles generated by Ad5/FC31 [35]. The simulations in [66] also show that in this type of concurrent mechanism, assembly of full shells is remarkably fast (two orders of magnitude faster than assembly of empty shells). Our results indicate that, when capsid-core assembly coupling is correct, AdV morphogenesis is a fast, highly cooperative process, as indicated by the scarcity of assembly intermediates in both virus variants studied, but most markedly in Ad5 wt. The Ad5/FC31 mutation changes shell-core interactions allowing us to catch assembly intermediates in a process otherwise too fast to be experimentally observed.

Because AdV has a dsDNA genome, encodes a putative packaging ATPase (IVa2), and produces large amounts of empty capsids, it seemed plausible that its assembly and packaging would take place in a sequential way similar to that of tailed phages. However, the organization of an AdV virion is quite different from that of a tailed phage. Instead, AdV belongs to a large group of viruses (the PRD1-adenovirus lineage) that use β-barrels orthogonal to the capsid surface to assemble icosahedral capsids with diameters ranging from ~0.06 to ~1 μm. The structural similarity found in the viral particles raised the question of a common ancestry for these viruses [67], which might also be reflected in aspects of their assembly mechanism.

Assembly and packaging in the smallest PRD1-AdV lineage viruses seem to follow different strategies depending on their particular genome type [68]. Bacteriophage PRD1 has a multi-protein channel spanning both capsid and membrane at a unique vertex, through which the linear genome is encapsidated into preformed capsid-membrane particles using power provided by its packaging ATPase [69, 70]. Conversely, the membrane-containing marine bacteriophage PM2 has a highly supercoiled circular genome, which would topologically complicate the motor driven DNA translocation. It has been proposed that interactions between the condensed PM2 genome and scaffolding membrane proteins start membrane bending and nucleate coat protein recruitment, coupling membrane morphogenesis, genome encapsidation, and capsid assembly [71]. The abundant coverage of the linear AdV genome by condensing proteins, producing a particularly tight complex in the immature particle [1, 38, 48–50, 72], would also pose a considerable topological complication for DNA translocation, which might be better resolved by a simultaneous assembly and packaging strategy.

Details consistent with the assembly and packaging mechanism proposed for PM2, which also bear striking similarities to our findings on AdV assembly, have been reported for some of the most complex PRD1-AdV lineage members. The Mimivirus factory originates from replication centers, as shown here for AdV [73]. Structural proteins are recruited to the viral membrane to create a first icosahedral vertex from which the capsid will grow. The open membrane, which holds the viral DNA, is progressively coated by capsid proteins to form an icosahedral particle, similarly to our observation of AdV capsid fragments engulfing the condensing core. A similar mechanism has been proposed for African swine fever virus (ASFV) [74]. Bacteriophage PM2, ASFV, and Mimivirus, all contain a membrane inside the icosahedral shell. In AdV, there is no internal membrane, but packaging protein L1 52/55 kDa may play a similar role to the membrane proteins in these viruses, helping to nucleate and keep together capsids fragments and growing cores during assembly.

Numerous questions remain to be answered to fully understand the AdV assembly mechanism. One of them is whether at least an initial, however imperfect, contact between capsid and core (*via* packaging proteins) would be necessary for capsids to start assembling. The existence of numerous packaging protein mutants that produce large amounts of empty capsids would indicate that this is not the case. However, a DBP thermo-conditional mutant with a 3-fold reduction in genome replication did not produce any kind of particles [21], and Ad5/FC31 incomplete particles had at least initiated packaging [75]. Since a variety of proteins (L1 52/55 kDa, IVa2, L4 33 kDa, IIIa) have a role in encapsidation, it is possible that deletion of only one of them still allows the initial stages of packaging, and therefore capsid assembly, to occur. The role of the packaging sequence Ψ is also intriguing. No empty particles were produced when part of the packaging sequence was deleted (mentioned in [64]), but an Ad5 construct containing loxP sites flanking the packaging sequence does accumulate empty particles when propagated in Cre-expressing cells [27, 28]. This observation does not rule out the concerted model, because deficient Cre function may result in incomplete cleavage of Ψ, still allowing an initial interaction between capsid and core components; also, it has been observed in the development of helper systems for gutless AdV production that residual helper virus can package genomes lacking Ψ [76]. Systematic molecular and structural characterization of assembly products from the various mutants would be required to assess this point.

Another unanswered question is whether the first capsid fragment to be recruited to the core would be chosen at random, or be defined by the presence of some “special feature”, similar to the stargate in Mimivirus [73]. Part of this special feature may be the presence of the putative packaging ATPase IVa2 [39]. It is an outstanding mystery what the role of the IVa2 ATPase function would be, if encapsidation does not happen by translocation through a portal. While packaging ATPases clustering in the HerA/FtsK superfamily are a common feature in PRD1-AdV lineage members [77], their exact function is not clear yet, and may vary in each particular virus. Additionally, IVa2 is an outlier, making up its own separate branch in the ATPase classification. In Mimivirus, it has been proposed that the ATPase may have a segregation-like role in disentangling individual genomes from the replication factory [78]. A similar role would be conceivable for IVa2 in our model.

In summary, we present here evidence that defines the location of the AdV assembly factory, and favors a concerted assembly and packaging mechanism for AdV morphogenesis. Detailed understanding of AdV assembly has been hindered by the lack of an *in vitro*, cell-free assembly system. The work presented here, together with the recently described isolation of functional replication centers [79], may constitute the basis for advancing towards such a system and conclusively resolve the dynamics of AdV morphogenesis.

## Methods

### Cells, viruses and antibodies

HEK293 cells (ATCC CRL-1573) were cultured at 37°C in Dulbecco’s modified Eagle’s medium (DMEM, Sigma Cat# D6429) supplemented with 2% fetal bovine serum (FBS, Biological Industries Cat# 04-001-1A), 10 units-10 μg/ml penicillin-streptomycin (Sigma Cat# P4333), 0.05 mg/ml gentamicin (Sigma Cat# G1397), 4 mM L-Glutamine (MERCK Cat# 3520) and 1X non-essential amino acid solution (Sigma Cat# M7145).

Control wild type virus was the Ad5 variant Ad5GL, where the E1 region has been deleted and substituted by GFP and firefly luciferase genes [80]. The Ad5/FC31 variant has an *attB/attP* insertion flanking Ψ and a GFP cassette following Ψ [59]. Virus propagation and purification was carried out as described [35].

The primary antibodies used were: rat anti BrdU monoclonal abcam #ab6326; rat anti Ad5 pVII serum [81]; mouse anti Ad2 IVa2 serum [82]; mouse anti Ad5 DBP monoclonal [83]; rabbit anti Ad5 L1 52/55 kDa serum [84]; and rabbit anti Ad5 fiber serum [85].

The secondary antibodies used were: for immunofluorescence assays, Alexa Fluor594 Goat Anti-Rat (Invitrogen #A-11007), Alexa Fluor555 Goat Anti-Rat (Invitrogen #A-21434), Pacific Blue Goat Anti-Mouse (Invitrogen #P-31582) and Pacific Blue Goat Anti-Rabbit (Invitrogen #P-10994). For immunoEM, 10 nm gold conjugated gold anti-rabbit (BB International #EM-GFAR10), 10 nm gold conjugated anti-mouse (EM-GFAF10), and 15 nm gold conjugated anti-rat (EM-GAT15).

### Conventional electron microscopy of infected cells

HEK293 cells grown in p100 culture plates to 70% confluence were infected with Ad5/FC31 or Ad5 wt with a multiplicity of infection (MOI) of 5. At the desired time post infection, the medium was removed and the cells were fixed with 2% glutaraldehyde and 1% tannic acid in 0.4 M HEPES pH 7.2 for 1.5 h at room temperature. Embedding in Epon was carried out as previously described [86]. Ultrathin sections (~70 nm) were collected on Formvar-coated nickel grids, stained with saturated uranyl acetate and lead citrate as described previously [86] and examined in a JEOL JEM 1230 transmission electron microscope at 100kV. Mock infected cells were processed in the same manner as a control.

### Immunofluorescence microscopy

Monolayer HEK293 cells grown in cover glasses were infected with Ad5 wt or Ad5/FC31 with MOI = 50. The infection was synchronized by incubating the cells for 30 min at 4°C and then 30 min at 37°C. Then, the inoculums were removed and medium was added. For BrdU labeling, after 18 h at 37°C the medium was changed by medium containing 25 μg/ml BrdU (5-Bromo-2’-deoxyuridine, Sigma Cat#B5002-1G), followed by another change at 25 hpi. Incubation with BrdU proceeded at 37°C. After 36 hpi, the medium was removed and 4% paraformaldehyde in PBS was added to the cells during 10 min. After 3 rinses with PBS, cover glasses were incubated with a mixture of 0.5% saponin and 10% FBS in PBS for 10 min. Samples were incubated with primary antibody in 0.5% saponin and 2% FBS in PBS during 45 min. After three more rinses, incubation with secondary antibodies diluted in 0.5% saponin and 2% FBS in PBS was carried out in darkness. After a final rinse with PBS, cover glasses were mounted on glass slides using ProLong (Invitrogen Cat# P36930) (4 μl drops). The antifade reagent was allowed to dry overnight before sample observation. All incubations were carried out at room temperature. Images were taken using a confocal multispectral Leica TCS SP5 system. Negative controls consisted of incubations without primary antibody, and immunolabeling of mock infected cells. In double labeling experiments, absence of cross-reactivity was verified by incubations with only one of the primary antibodies and the secondary antibody corresponding to the other.

The following modifications to the protocol described above were applied for anti-BrdU labeling: fixed samples were washed three times with saponin 1% in PBS (3x5 min), then subjected to DNA denaturing treatment: 1N HCl during 10 min at 4°C, followed by 2N HCl during 10 min at room temperature, and finally 20 min at 37°C. To neutralize, borate buffer (4 g NaOH; 23.5 g boric acid to 500 ml in milliQ water, pH 8.2) was added for 12 min at room temperature, and the protocol continued with the block-permeabilization step described above, but with 1% instead of 0.5% saponin. For double labeling assays, the primary antibodies were used together in the same incubation, except for the case of double labeling for IVa2 and L1 52/55 kDa. In this case, samples were incubated with the mouse anti-IVa2 serum and fixed with 4% paraformaldehyde for 5 min before incubating with the rabbit anti-L1 52/55 kDa serum. Immunofluorescence image analyses were carried out with Image J [87].

### Freeze-substitution and immunoelectron microscopy

BrdU labeling of newly synthesized DNA was carried out as described for immunofluorescence, with HEK293 cells grown in p100 culture plates instead of cover glasses. Infected and control cells were fixed with 4% paraformaldehyde in PBS. After rinsing three times with PBS, glycerol was added drop by drop to 15% final concentration. After 15 min at 4°C, glycerol was increased to 30% and 15 min later the cells were harvested, pelleted and frozen by plunge freezing in liquid ethane using a Leica CPC plunger. Freeze substitution was carried out in a Leica EM automatic freeze-substitution system (AFS). Samples were maintained in 0.5% uranyl acetate in dry methanol for 60 h at -90°C, with several changes of dehydrating solution. Then, the temperature was raised in a controlled manner to reach -40°C after 7 h and maintained to the end of the procedure. Samples were rinsed with dry methanol, and infiltrated with growing concentrations of Lowicryl HM20 in methanol for 24 h. Polymerization was carried out by UV irradiation for 48 h at -40°C, and then 48 h at 20°C. Ultrathin sections were obtained as described in the conventional electron microscopy section.

For immunoEM assays, grids carrying freeze-substituted ultrathin sections were placed on TBG (30 mM Tris-HCl pH 8, 150 mM NaCl, 0.1% BSA and 1% gelatin) drops for 10 min, incubated with the primary antibody in TBG for 30 min, washed 3 times with PBS, floated on 4 TBG drops (5 min per drop) and incubated in gold-conjugated secondary antibodies diluted in TBG for 30 min. Then, the grids were washed 3 times with PBS and milli-Q water, and stained with saturated uranyl acetate [86]. All incubations were carried out at room temperature. Negative controls consisted of incubations without primary antibody, and immunolabeling of mock infected cells.

For anti-BrdU labeling, an additional step was required. Sections were treated with 0.2 mg/ml proteinase K (Roche Cat# 3115879) for 15 min at 37°C, then washed with milli-Q water and denatured with 2N HCl for 25 min. After several (~4) rinses in milli-Q water, the protocol continued with the TBG incubation. To unmask protein VII, sections were floated on three drops of DNase buffer (10 mM Tris-HCl pH 8.2, 10 mM NaCl, 5 mM MgCl_2_) (5 min per drop), then incubated with 50 μg/ml DNAse (Sigma Cat# D5025) for 1 hour at 37°C, rinsed in milli-Q water and transferred to TBG to start the immunogold labeling protocol. Incubation with the anti-VII primary antibody was performed overnight at 4°C.

### Statistical analyses

To quantify the occurrence of structural features (EOGs, SBs, assembly intermediates) or gold labels in Epon embedded or FS samples, sections were scanned at low magnification to locate cells with replication centers, which were then imaged at high magnification in as many micrographs as needed to cover all the visible part of the nucleus. The area corresponding to the different nuclear regions (PRZ, DAS, rest of the nucleus) was measured using ImageJ [87]. Gold labels in viral particles were not included in the quantification. Data are presented as box plots where center lines show the medians; box limits indicate the 25th and 75th percentiles; and whiskers extend 1.5 times the interquartile range from the 25th and 75th percentiles. Details of statistical analyses are summarized in **S1 Table**.

## Supporting information

S1 Text Additional Details on the Localization of AdV Packaging Proteins. (DOCX)

S1 Table Results of statistical tests to assess the significance of differences between datasets used in this study. (DOCX)

S1 Fig Shutoff of cell DNA synthesis upon infection by Ad5 wt or Ad5/FC31. HEK 293 cells were infected, fixed at the indicated times post-infection and labeled for BrdU as described in Methods. One hour previous to fixation the medium was replaced by new medium containing 25 μg/ml BrdU. A sharp decrease in BrdU signal correlates with the expected shutoff of cellular DNA synthesis at ~18 hpi [62] for both Ad5 wt and Ad5/FC31. Imaging conditions were the same for all samples. Notice the weak GFP signal due to the harsh treatment required for BrdU labeling. Scale bar: 20 μm.
(TIF)

S2 Fig AdV replication centers at late times post-infection observed by conventional electron microscopy. Sections of cells infected with Ad5 wt (**A-C**) or Ad5/FC31 (**D-G**) at 36 hpi (MOI = 5). Contours in **B** and **E** indicate the possible PRZ (magenta) and DAS (green) regions. The contour colors are chosen for comparison with the BrdU/DBP double labeling shown in Fig 1D. (**C, F)** Zoom of the areas highlighted by white dashed rectangles in **A** and **D**. (**G)** Section of a cell infected with Ad5/FC31 at 48 hpi (MOI = 5). EOGs are interspersed in loose electron-dense material suggestive of DNA by its texture (DNA bundles, b). Numerous viral particles are also present. Nucleus (**N**); cytoplasm (**C**); lobes (**lo**); electron-opaque grains (**eogs**); interchromatin granules (**ig**). White arrows indicate EOGs. Scale bar in **A, B**,**D and E**, 1 μm. In **C**, **F** and **G**, 500 nm.
(TIF)

S3 Fig Ad5 wt and Ad5/FC31 produce structures positive for packaging and capsid proteins but not for core proteins or viral genomes. **(A)** Double labeling against L1 52/55 kDa and IVa2 in Ad5 wt infected cells (MOI = 50, 36 hpi). Images in the leftmost column show a general view of sample, while the areas highlighted by white dotted squares are shown at larger magnification in the other columns. **1.** Clusters with only L1 52/55 kDa signal. **2.** Clusters with both L1 52/55 kDa and IVa2 signal. **3.** Small rings positive for both L1 52/55 kDa and IVa2. Scale bar: 5 μm for the leftmost column, 2 μm for the rest. Arrows point to label in clusters. (**B)** Electron-dense inclusions (left hand side columns) and small rings (right hand side columns) produced by wt and Ad5/FC31, observed by freeze-substitution and electron microscopy and labeled against L1 52/55 kDa, IVa2 or fiber, as indicated. For IVa2 labeling, sections were treated with DNase before immunolabeling, in an attempt to unmask IVa2 epitopes. Scale bars: 200 nm.
(TIF)

S4 Fig Localization of protein IVa2 by immunoelectron microscopy. HEK293 cells infected with Ad5 wt (**A and C**) or Ad5/FC31 (**B and D**) at 48 hpi. Green area: DAS. Magenta area: PRZ. (**A, B)** General view of replication centers. (**C, D)** Virus particles and DNA bundles (b). Sections were treated with DNase before immunolabeling, in an attempt to unmask protein IVa2 epitopes. All arrows indicate the presence of gold particles; closed black arrows and black arrowheads specify the presence of IVa2 in DNA bundles and EOGs respectively. Scale bars: **A** and **B,** 300 nm; **C** and **D**; 100 nm. **(E)** Quantification of label for polypeptide IVa2 in the different nuclear regions of infected cells.
(TIF)

S5 Fig EOGs are more abundant in Ad5/FC31 than in wt Ad5 infections. Epon embedded cells infected with Ad5 wt **(A, B)** or Ad5/FC31 **(C, D)**. MOI = 5, 24 hpi. (**B, D)** Zoom in areas within the black dotted rectangles in (A) and (C). White arrows indicate the presence of EOGs. Green area: DAS. Magenta area: PRZ. Virus particles (v); nucleoplasm (np). Scale bars, 500 nm. **(E)** Quantification of EOG abundance. The number of EOGs per unit area in Epon-embedded cells at 48 hpi is shown.
(TIF)
