# Supplementary material for: Localization of adenovirus morphogenesis players, together with visualization of assembly intermediates and failed products, favor a model where assembly and packaging occur concurrently at the periphery of the replication center
Source: PLoS Pathog. 2017 Apr 27;13(4):e1006320. doi: 10.1371/journal.ppat.1006320 (PMC5409498; doi:10.1371/journal.ppat.1006320)
Supplement: S1 Text — (DOCX) [file ppat.1006320.s001.docx]

**S1 Text. Additional Details on the Localization of AdV Packaging Proteins.**

In single labeling experiments, no difference was observed between wt and Ad5/FC31 infections for the L1 52/55 kDa or IVa2 inmunofluorescence labeling pattern. Both proteins showed diffuse signal around unlabeled areas in the nucleus, as shown in **Fig. 1** in the main text for L1 52/55 kDa. When combined with BrdU labeling, no signal for IVa2 was observed, indicating that the reactivity of the anti-IVa2 antibodies is sensitive to the acid treatment required to label DNA with BrdU. Label for both L1 52/55 kDa and IVa2 was also found in clusters and small rings (maximum diameter 1.6 ± 0.3 µm, n = 37), sometimes simultaneously (**S3A Fig**). However, these structures were not positive for BrdU, and therefore could not represent packaging sites.

In FS, signal for packaging protein IVa2 was observed scattered throughout the nucleus, but was practically absent from the DAS (**S4A,B Fig**). IVa2 was not easily detected in viral particles (**S4C,D Fig**): only a few capsids had signal. This result is consistent with the protein low copy number (~5) and highly localized position at a single vertex [[1](#_ENREF_1), [2](#_ENREF_2)]. There would be very little chance of exposing just the adequate vertex in a resin section.

Abundant label for both L1 52/55 kDa and IVa2 was also found in electron-dense, smooth inclusions outside the PRZ (**S3B Fig**), which by size (diameter 1.1 µm ± 0.4 µm, n=16) and amount of label could correspond to the clusters observed by immunofluorescence. However, as mentioned previously, these substructures were not positive for BrdU. Smooth electron-dense inclusions were also positive for fiber, but not for core protein VII (**S3B Fig**). We interpret this observation as a sign that these inclusions are deposits for excess or misfolded viral proteins. Similar structures are produced by aggregated components in a Vaccinia virus mutant that fails to properly package the viroplasm [[3](#_ENREF_3)]. No label for either of the packaging proteins tested was detected in ring-like structures, even in compact rings, which had previously been reported to contain protein IVa2 [[4](#_ENREF_4)] and were obvious candidates by shape and size (**S3B Fig**). Therefore, it is not possible at this point to correlate the small rings positive for both IVa2 and L1 52/55 kDa in immunofluorescence assays with any structure observed by immunoEM.

**Supporting References**

1. Christensen JB, Byrd SA, Walker AK, Strahler JR, Andrews PC, Imperiale MJ. Presence of the adenovirus IVa2 protein at a single vertex of the mature virion. Journal of virology. 2008;82(18):9086-93. PubMed PMID: 18614642.

2. Benevento M, Di Palma S, Snijder J, Moyer CL, Reddy VS, Nemerow GR, et al. Adenovirus composition, proteolysis, and disassembly studied by in-depth qualitative and quantitative proteomics. The Journal of biological chemistry. 2014;289(16):11421-30. doi: 10.1074/jbc.M113.537498. PubMed PMID: 24591515; PubMed Central PMCID: PMC4036278.

3. Condit RC, Moussatche N. The vaccinia virus E6 protein influences virion protein localization during virus assembly. Virology. 2015;482:147-56. doi: 10.1016/j.virol.2015.02.056. PubMed PMID: 25863879; PubMed Central PMCID: PMCPMC4461454.

4. Lutz P, Puvion-Dutilleul F, Lutz Y, Kedinger C. Nucleoplasmic and nucleolar distribution of the adenovirus IVa2 gene product. Journal of virology. 1996;70(6):3449-60. Epub 1996/06/01. PubMed PMID: 8648677; PubMed Central PMCID: PMC190218.
