# Supplementary material for: Localization of adenovirus morphogenesis players, together with visualization of assembly intermediates and failed products, favor a model where assembly and packaging occur concurrently at the periphery of the replication center
Source: PLoS Pathog. 2017 Apr 27;13(4):e1006320. doi: 10.1371/journal.ppat.1006320 (PMC5409498; doi:10.1371/journal.ppat.1006320)
Supplement: S1 Table — (DOCX) [file ppat.1006320.s002.docx]

**S1 Table**. **Results of statistical tests to assess the significance of differences between datasets used in this study.** The Mann-Whitney Rank Sum Test (**MW**) was used instead of the two-tailed t-test (**2T**) when the data failed the Shapiro-Wilk Normality Test. All tests were run with SigmaPlot (Systat Software, Inc.). **NS**: no statistically significant difference; *****: P<0.05; ******: P<0.01; *******: P<0.005. The tests relate to plots shown in the figure indicated in the rightmost column. Cells related to Ad5 **wt** virus are shadowed in **blue**, and cells related to the Ad5/**FC31** mutant in **orange**, to conserve the color scheme used in boxplots. **N** indicates the number of experiments or cells analyzed, and the section area scanned for gold particle or EOG counting.

|  |  | **1st dataset to compare** | **2nd dataset to compare** | **Test** | **Result** | **N** | **Figure** |
| --- | --- | --- | --- | --- | --- | --- | --- |
| **Percentage of BrdU labeled cells (immunofluorescence)** | | | | | | | |
|  |  | wt | FC31 | 2T | *  P=0.020 | wt:  4 experiments, 116 cells.  FC31:  5 experiments, 112 cells. | 1 |
| **Number of EOGs per µm^2^ of PRZ in EPON-embedded samples** | | | | | | | |
|  |  | wt | FC31 | MW | ***  P<0.001 | wt:  36 cells,  663 µm^2^.  FC31:  45 cells,  741 µm^2^. | S5 |
| **Gold labels per µm^2^ in different nuclear regions in cells infected with either Ad5 wt or Ad5/FC31 (immunoelectron microscopy)** | | | | | | | |
| **Label for** | **Virus** |  |  |  |  |  |  |
| **BrdU** | wt | PRZ | DAS | MW | ***  P<0.001 | 13 cells,  2003 µm^2^ | 2 |
|  |  | PRZ | other | MW | ***  P<0.001 |  |  |
|  |  | DAS | other | MW | NS  P=0.117 |  |  |
|  | FC31 | PRZ | DAS | MW | ***  P<0.001 | 13 cells,  1658 µm^2^ |  |
|  |  | PRZ | other | MW | ***  P<0.001 |  |  |
|  |  | DAS | other | MW | NS  P=1.000 |  |  |
|  |  |  |  |  |  |  |  |
| **L1 52/55 kDa** | wt | PRZ | DAS | MW | *  P=0.015 | 10 cells,  921 µm^2^ | 3 |
|  |  | PRZ | other | MW | NS  P=0.111 |  |  |
|  |  | DAS | other | MW | *  P=0.011 |  |  |
|  | FC31 | PRZ | DAS | 2T | ***  P<0.001 | 10 cells,  793 µm^2^ |  |
|  |  | PRZ | other | MW | NS  P=0.427 |  |  |
|  |  | DAS | other | MW | ***  P<0.001 |  |  |
|  |  | SB | PRZ | 2T | NS  P=0.561 |  |  |
|  |  | SB | DAS | 2T | ***  P<0.001 |  |  |
|  |  | SB | other | 2T | NS  P=0.379 |  |  |
|  |  |  |  |  |  |  |  |
| **IVa2** | wt | PRZ | DAS | MW | ***  P<0.001 | 10 cells,  1299 µm^2^ | S4 |
|  |  | PRZ | other | MW | NS  P=0.212 |  |  |
|  |  | DAS | other | MW | ***  P<0.001 |  |  |
|  | FC31 | PRZ | DAS | MW | ***  P<0.001 | 10 cells,  1167 µm^2^ |  |
|  |  | PRZ | other | 2T | ***  P=0.003 |  |  |
|  |  | DAS | other | 2T | ***  P<0.001 |  |  |
|  |  |  |  |  |  |  |  |
| **VII** | wt | PRZ | DAS | MW | ***  P<0.001 | 13 cells,  2360 µm^2^ | 4 |
|  |  | PRZ | other | MW | ***  P<0.001 |  |  |
|  |  | DAS | other | MW | ***  P=0.002 |  |  |
|  | FC31 | PRZ | DAS | MW | ***  P<0.001 | 15 cells,  1043 µm^2^ |  |
|  |  | PRZ | other | MW | ***  P<0.001 |  |  |
|  |  | DAS | other | MW | *  P=0.012 |  |  |
|  |  | SB | PRZ | 2T | NS  P=0.722 |  |  |
|  |  | SB | other | MW | ***  P=0.001 |  |  |
|  |  | SB | DAS | MW | ***  P<0.001 |  |  |
|  |  |  |  |  |  |  |  |
|  |  |  |  |  |  |  |  |
| **Fiber** | wt | PRZ | DAS | 2T | ***  P=0.003 | 8 cells,  755 µm^2^ | 5 |
|  |  | PRZ | other | MW | NS  P=0.328 |  |  |
|  |  | DAS | other | MW | **  P=0.009 |  |  |
|  | FC31 | PRZ | DAS | 2T | ***  P=0.003 | 8 cells,  831 µm^2^ |  |
|  |  | PRZ | other | MW | **  P=0.007 |  |  |
|  |  | DAS | other | 2T | ***  P<0.001 |  |  |
|  |  | SB | PRZ | 2T | NS  P=0.878 |  |  |
|  |  | SB | DAS | MW | *  P=0.043 |  |  |
|  |  | SB | other | 2T | ***  P<0.001 |  |  |
|  |  |  |  |  |  |  |  |
| **VII** | FC31 | SB (**no DNase)** | SB (**after** **DNase)** | 2T | *  P=0.0231 | 15 cells in each case | 7 |
|  |  |  |  |  |  |  |  |
