# Supplementary figures and images for: Localization of adenovirus morphogenesis players, together with visualization of assembly intermediates and failed products, favor a model where assembly and packaging occur concurrently at the periphery of the replication center
Source: PLoS Pathog. 2017 Apr 27;13(4):e1006320. doi: 10.1371/journal.ppat.1006320 (PMC5409498; doi:10.1371/journal.ppat.1006320)

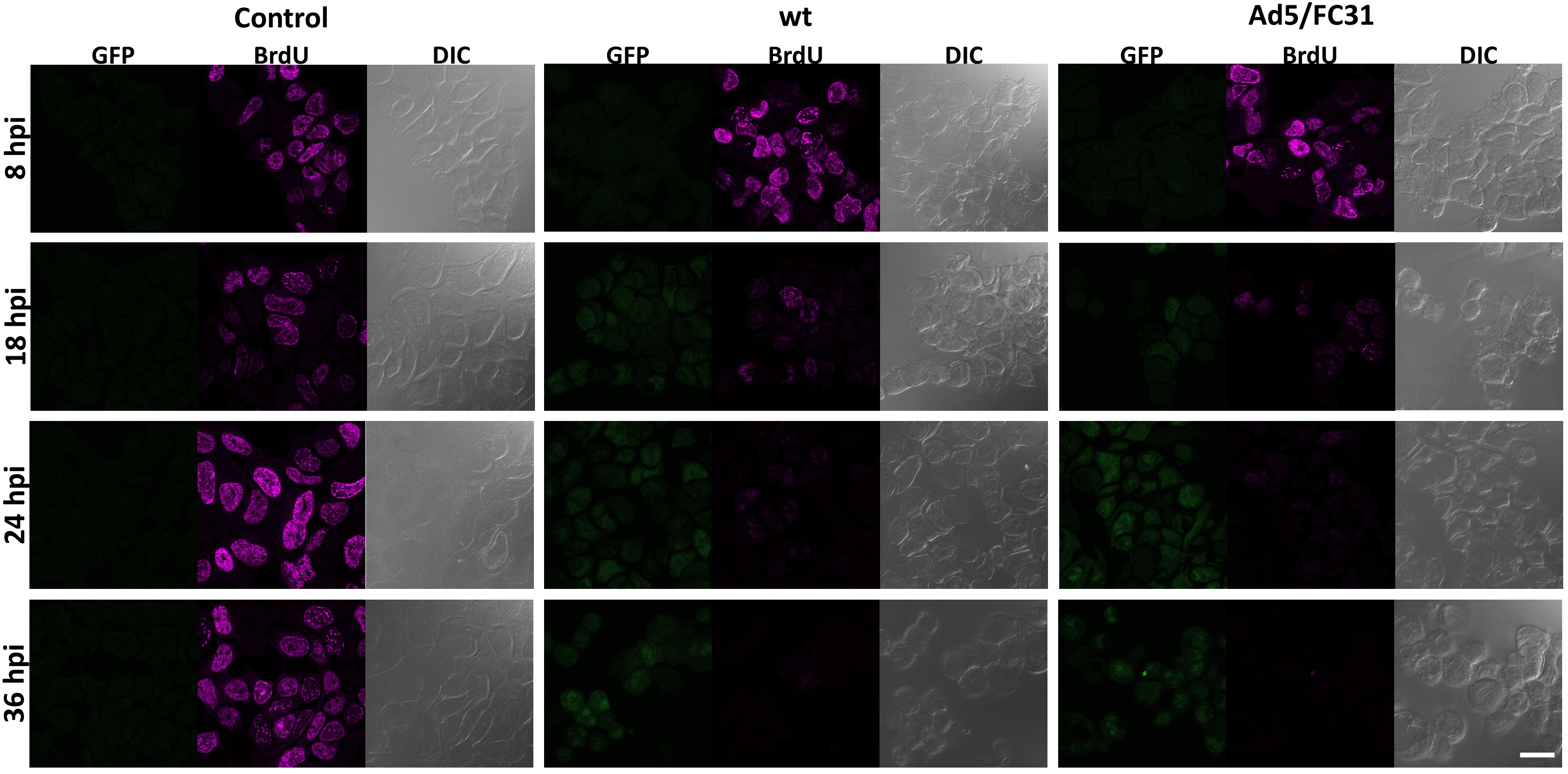

Supplement: S1 Fig — HEK 293 cells were infected, fixed at the indicated times post-infection and labeled for BrdU as described in Methods. One hour previous to fixation the medium was replaced by new medium containing 25 μg/ml BrdU. A sharp decrease in BrdU signal correlates with the expected shutoff of cellular DNA synthesis at ~18 hpi [62] for both Ad5 wt and Ad5/FC31. Imaging conditions were the same for all samples. Notice the weak GFP signal due to the harsh treatment required for BrdU labeling. Scale bar: 20 μm. (TIF) [file ppat.1006320.s003.tif]

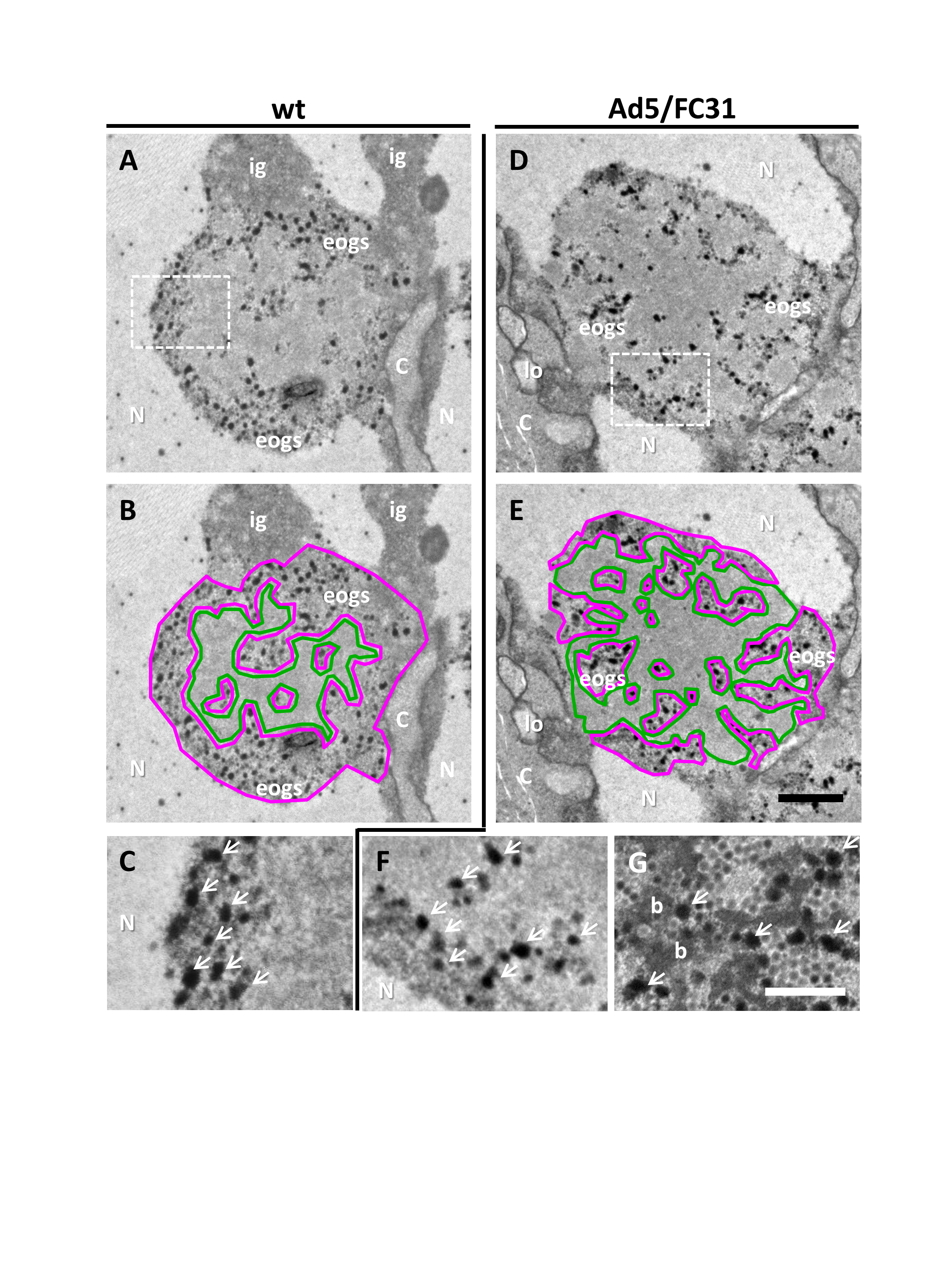

Supplement: S2 Fig — Sections of cells infected with Ad5 wt (A-C) or Ad5/FC31 (D-G) at 36 hpi (MOI = 5). Contours in B and E indicate the possible PRZ (magenta) and DAS (green) regions. The contour colors are chosen for comparison with the BrdU/DBP double labeling shown in Fig 1D. (C, F) Zoom of the areas highlighted by white dashed rectangles in A and D. (G) Section of a cell infected with Ad5/FC31 at 48 hpi (MOI = 5). EOGs are interspersed in loose electron-dense material suggestive of DNA by its texture (DNA bundles, b). Numerous viral particles are also present. Nucleus (N); cytoplasm (C); lobes (lo); electron-opaque grains (eogs); interchromatin granules (ig). White arrows indicate EOGs. Scale bar in A, B,D and E, 1 μm. In C, F and G, 500 nm. (TIF) [file ppat.1006320.s004.tif]

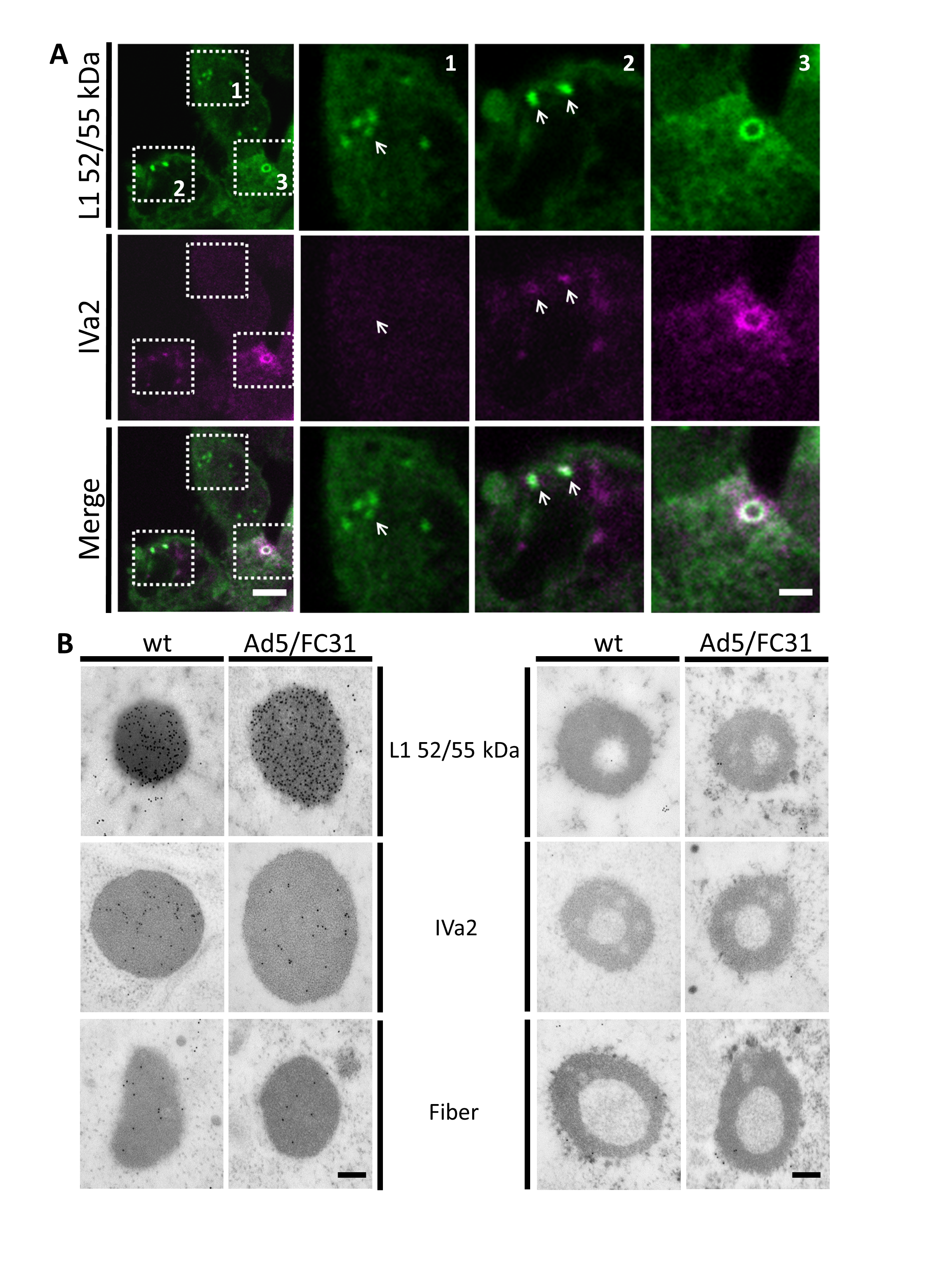

Supplement: S3 Fig — (A) Double labeling against L1 52/55 kDa and IVa2 in Ad5 wt infected cells (MOI = 50, 36 hpi). Images in the leftmost column show a general view of sample, while the areas highlighted by white dotted squares are shown at larger magnification in the other columns. 1. Clusters with only L1 52/55 kDa signal. 2. Clusters with both L1 52/55 kDa and IVa2 signal. 3. Small rings positive for both L1 52/55 kDa and IVa2. Scale bar: 5 μm for the leftmost column, 2 μm for the rest. Arrows point to label in clusters. (B) Electron-dense inclusions (left hand side columns) and small rings (right hand side columns) produced by wt and Ad5/FC31, observed by freeze-substitution and electron microscopy and labeled against L1 52/55 kDa, IVa2 or fiber, as indicated. For IVa2 labeling, sections were treated with DNase before immunolabeling, in an attempt to unmask IVa2 epitopes. Scale bars: 200 nm. (TIF) [file ppat.1006320.s005.tif]

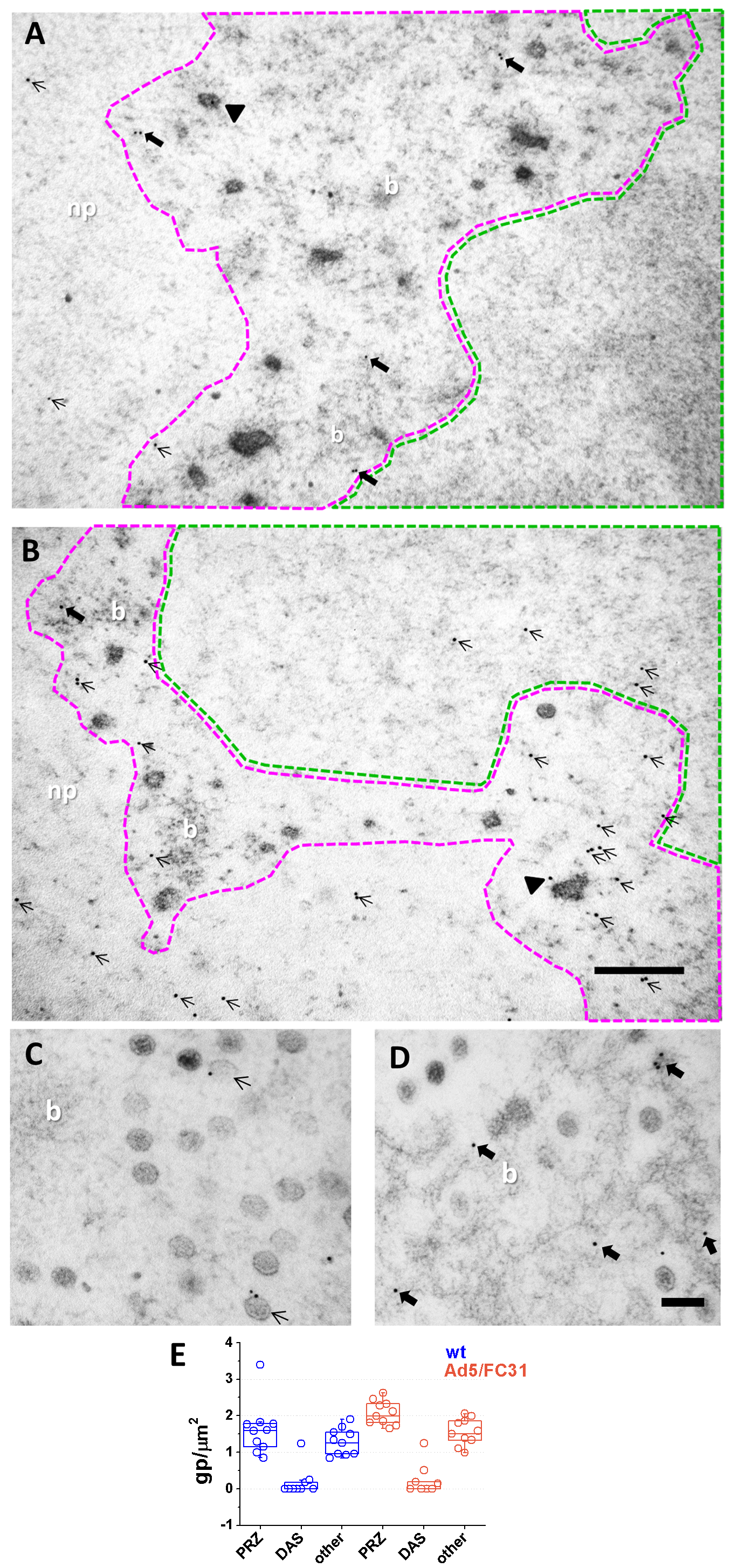

Supplement: S4 Fig — HEK293 cells infected with Ad5 wt (A and C) or Ad5/FC31 (B and D) at 48 hpi. Green area: DAS. Magenta area: PRZ. (A, B) General view of replication centers. (C, D) Virus particles and DNA bundles (b). Sections were treated with DNase before immunolabeling, in an attempt to unmask protein IVa2 epitopes. All arrows indicate the presence of gold particles; closed black arrows and black arrowheads specify the presence of IVa2 in DNA bundles and EOGs respectively. Scale bars: A and B, 300 nm; C and D; 100 nm. (E) Quantification of label for polypeptide IVa2 in the different nuclear regions of infected cells. (TIF) [file ppat.1006320.s006.tif]

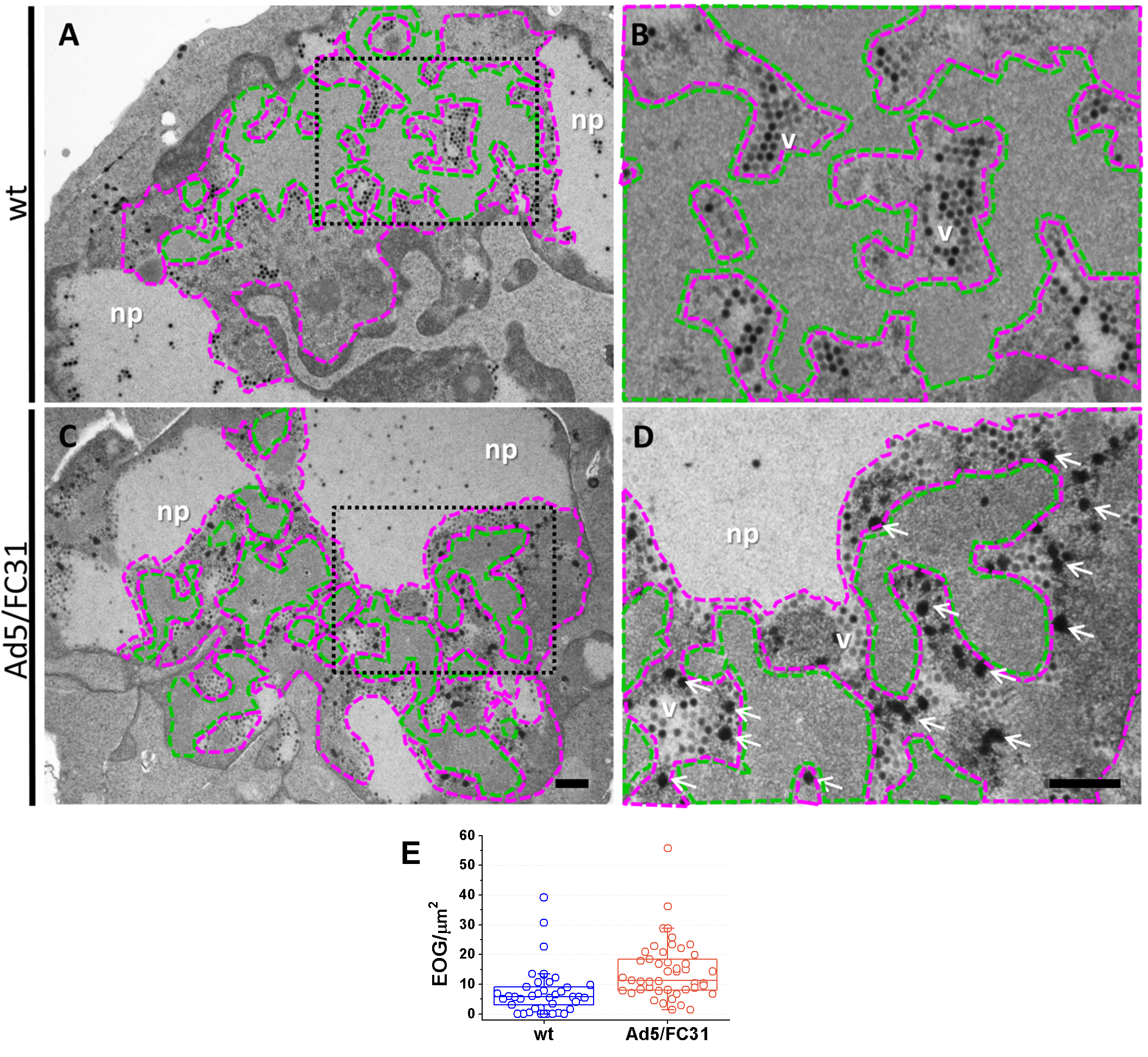

Supplement: S5 Fig — Epon embedded cells infected with Ad5 wt (A, B) or Ad5/FC31 (C, D). MOI = 5, 24 hpi. (B, D) Zoom in areas within the black dotted rectangles in (A) and (C). White arrows indicate the presence of EOGs. Green area: DAS. Magenta area: PRZ. Virus particles (v); nucleoplasm (np). Scale bars, 500 nm. (E) Quantification of EOG abundance. The number of EOGs per unit area in Epon-embedded cells at 48 hpi is shown. (TIF) [file ppat.1006320.s007.tif]
